# Protocol for blastomere injection to generate bilateral hemimosaic *Xenopus* tadpoles

**DOI:** 10.1016/j.xpro.2026.104387

**Published:** 2026-02-19

**Authors:** Vanessa J. Li, Philip M. Kesner, Anne Schohl, David Foubert, Nicholas Benfey, Edward S. Ruthazer

**Affiliations:** 1 Montreal Neurological Institute-Hospital, McGill University, Montreal, Quebec H3A 2B4, Canada

**Keywords:** developmental biology, microscopy, model organisms, neuroscience

## Abstract

Here, we present a protocol to generate bilateral hemimosaic *Xenopus* tadpoles that allows systematic, independent manipulation of pre- and postsynaptic neurons in the tadpole retinotectal projection. We describe the steps to perform *in vitro* fertilization, followed by blastomere injections for two different manipulations. Specifically, we detail procedures for exogenous gene expression (GCaMP6s) by mRNA microinjection and endogenous gene knockdown (GluN1) with antisense morpholino microinjection.

For complete details on the use and execution of this protocol, please refer to Kesner et al.^1^ and Li et al.^2^

## Highlights

• Steps for preparation of mRNA and MO solutions for blastomere injection
• Procedures for collection and *in vitro* fertilization of *Xenopus* eggs
• Instructions for blastomere injection of mRNA or MO
• Guidance on screening hemimosaic animals resulting from blastomere injections

## Abstract (editor highlights)

Publisher’s note: Undertaking any experimental protocol requires adherence to local institutional guidelines for laboratory safety and ethics.

## Graphical abstract

**Figure:**
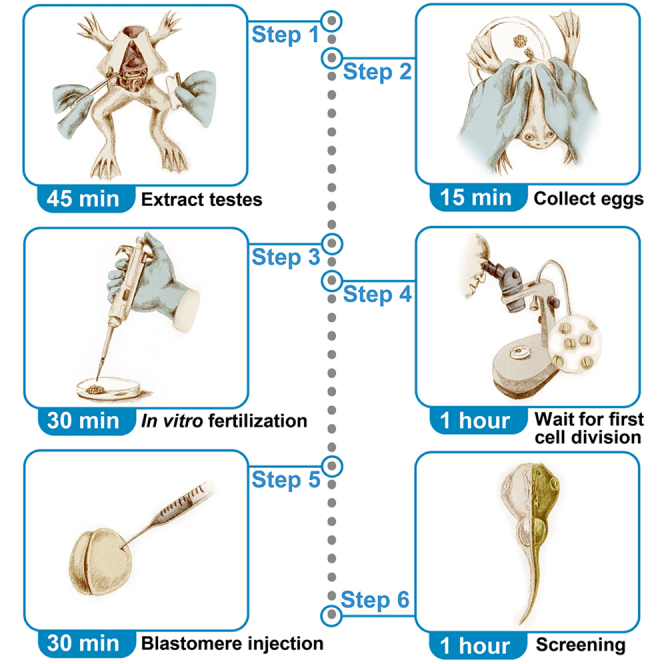

## Before you begin

The retinotectal system of the *Xenopus laevis* tadpole is a powerful experimental model for studying development of the nervous system, popular for its accessibility and robustness to a wide range of experimental manipulations.^3^^,^^4^^,^^5^ Xenopus develop externally from large oocytes, making them easily accessible for genetic and pharmacological interventions from very early developmental stages. Albino Xenopus tadpoles have transparent skin, allowing their brain structures to be imaged *in vivo* without surgical preparation. Targeted manipulation of retinal ganglion cell (RGC) axons or their postsynaptic tectal neurons in the retinotectal circuit has revealed important principles in neural circuit connectivity and function.^6^^,^^7^ However, commonly applied techniques such as viral infection,^8^^,^^9^^,^^10^ electroporation^11^^,^^12^ and lipofection^13^^,^^14^ can only crudely target individual or subsets of neurons in the retinotectal neuronal population. Gal4-UAS and Cre-loxP transgenic systems have been used successfully to generate cell-type and region-specific gene alteration in rodent and zebrafish models,^15^^,^^16^^,^^17^ but this technique is time-consuming to implement in Xenopus laevis, which has a relatively protracted period of metamorphosis to reach sexual maturity, and to date there is a general lack of Cre lines available for the Xenopus model. Hence, a method that can systematically and separately manipulate pre- and postsynaptic neurons in the Xenopus retinotectal circuit has the potential to open uncharted avenues in developmental research in Xenopus and offer new insights into neurodevelopment at the whole circuit level.

**Figure 1:**
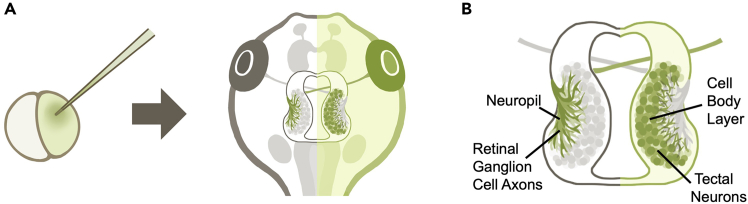
Hemimosaic genetic manipulation through blastomere injection (A) Injecting mRNA or morpholino oligonucleotide into one blastomere of two-cell stage tadpole embryos results in mosaic expression of protein or morpholino restricted to one lateral half of the body. (B) Schematic of the tadpole retinotectal system. The tadpole tectum can be visually separated into two regions: the cell body layer mainly consisting of somata of tectal cells, and the neuropil layer mainly consisting of the dendrites of tectal neurons and the axon terminals of RGCs projecting in from the contralateral eye. Due to the crossing over of RGC axons, tadpoles with hemimosaic expression of fluorescent protein or morpholino will display fluorescent protein expression or morpholino knockdown in RGC axon terminals in one tectal hemisphere, and in tectal neurons in the opposite hemisphere.

Microinjection into one blastomere of a two-cell stage tadpole embryo results in spatially restricted promulgation of the injected material within one lateral half of the animal as it develops. Since RGC axons in Xenopus project almost exclusively to the contralateral tectal hemisphere,^18^ the injected material will be present only in postsynaptic tectal cells in one tectal hemisphere and in the presynaptic RGC axonal inputs in the opposite hemisphere, effectively targeting the pre- and postsynaptic components of the retinotectal circuit in each hemisphere respectively (Figure 1).

We have used this injection technique with antisense Morpholino oligonucleotides (MO) to generate tadpoles with hemimosaic knockdown of the GluN1 subunit of the N-methyl-D-aspartate receptor (NMDAR), demonstrating distinct roles for NMDARs in the RGCs and tectal neurons for circuit development.^1^ MOs are synthetic DNA analogues with morpholine rings instead of deoxyribose sugar and phosphorodiamidate links instead of phosphates, rendering them more stable against degradation by nucleases and proteases. Antisense MOs can inhibit gene expression by binding complementary RNA sequences.

Using mRNA microinjection, we also produced tadpoles with hemimosaic expression of the genetically encoded calcium indicator GCaMP6s.^2^^,^^19^ GCaMP is a genetically-encoded Ca^2+^ indicator that can be used to visualize neuronal activity, as well as other processes that raise intracellular Ca^2+^ levels. We have recently used this method to reveal the emergence of functional pre- and postsynaptic topographic maps in the Xenopus visual system.^2^ Microinjection of mRNA can also be used to express a large variety of other genetically-encoded sensors without requiring the time-consuming process of generating transgenic lines.

In this protocol, we describe the steps for GluN1 knockdown with antisense MO and for GCaMP6s expression with mRNA blastomere injection. Both manipulations share the same procedures for *in vitro* fertilization and blastomere injection, differing only in the preparation of the injected material. These examples demonstrate the practical use of the blastomere injection framework for targeted visualization and genetic manipulation of pre- and postsynaptic components of a neural circuit. This protocol can be easily applied to generate animals with hemimosaic knockdown of other target proteins (via MO injection, CRISPR/cas9 injection, etc.), or hemimosaic expression of other proteins, such as exogenous optical indicators and dominant negative or constitutively active mutant genes of interest.

### Innovation

Generation of bilateral mosaic animals by microinjection of mRNA, MO or CRISPR/cas9 reagents at the two-cell stage has been used to provide within-animal controls for genetic manipulations in early embryogenesis.^20^ In later development, hemimosaicism offers additional advantages for studying neural circuit formation and function that have yet to be fully exploited. In particular, the ability to target manipulations to contralaterally projecting axons or their target cells in the brain is a powerful tool for dissection of neuronal function. Here we demonstrate applications in later stage hemimosaic tadpoles to study neuronal circuit formation in the visual system, taking advantage of the contralaterally-projecting retinotectal projection to independently manipulate and visualize presynaptic RGC axon inputs and postsynaptic retinorecipient tectal cells in the same animal.^1^^,^^2^ This method allows for rapid introduction and testing of novel functional indicators in vivo for use in the F0 generation, saving time and effort compared to transgenesis. This protocol will be valuable as an integrated resource for systems neuroscientists whose research is focused on functional neural circuit development.

### Institutional permissions

Because these procedures effectively result in the production of genetically modified organisms, they should be treated as such when applying for approval from local institutional animal use committees, including descriptions of the anticipated impacts on the full organism. All procedures in this paper were approved by the Animal Care Committee of the Montreal Neurological Institute at McGill University and carried out in accordance with Canadian Council on Animal Care guidelines.

**Figure 2:**
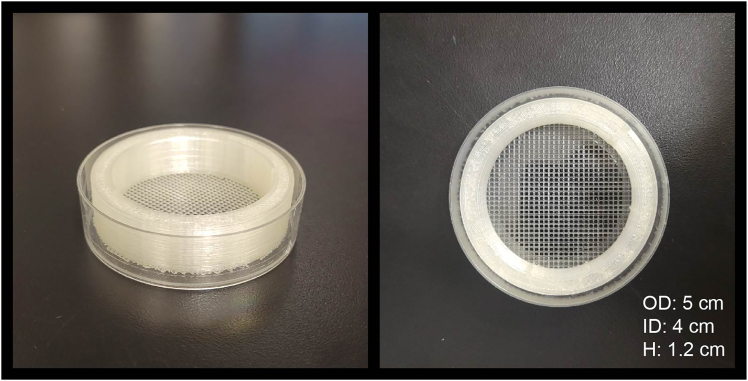
3D-printed injection chamber placed inside a 60 cm diameter Petri dish The outer diameter (OD), inner diameter (ID) and height (H) of the injection chamber is 5 cm, 4 cm and 1.2 cm, respectively. The grid spacing of the bottom mesh is 1.2 mm.

### Preparation of solutions and the blastomere injection chamber

**Timing: 3 h**

1. Prepare the following solutions:
  a. 0.1× modified Barth’s solution with HEPES buffer (MBSH) for rearing animals.
  b. 1× MBSH for storing male frog testes and preparing Ficoll solution.
  c. 0.2% MS-222 in 0.1× MBSH for tadpole and frog euthanasia.
  d. 0.02% MS-222 in 0.1× MBSH for tadpole anaesthesia.
  e. 2% Ficoll in 1× MBSH for blastomere injection.
  f. 1% Ficoll in 0.1× MBSH for embryo incubation.

***Note:*** For countries where MS-222 is not permitted for adult euthanasia, intracoelomic pentobarbital (60 to 100 mg/kg body weight) can be used.

2. Prepare the embryo injection chamber (Figure 2).

***Note:*** The injection chamber is composed of a plastic ring designed to fit inside a 60 mm diameter petri dish, with a square mesh with 1.2–1.4 mm spacing attached to the bottom to line up eggs for injection. The chamber can be 3D printed with the 3D print file provided at https://github.com/RuthazerLab/Hemimosaic-3d-models. Print the part with a raft with settings of 1.2–1.4 mm spacing and 0.8 mm thickness to produce the bottom mesh. Use PLA or an equivalent nontoxic filament.

### Prepare mRNA or MO solutions for blastomere injections

**Timing: 1–2 days**

3. For expressing GCaMP6s using mRNA injections, prepare GCaMP6s mRNA.
  ***Note:*** GCaMP6s needs to be subcloned into pCS2+ vector for transcription into RNA or any other vector containing a SP6 RNA promotor and a SV40 polyA sequence.
  **CRITICAL:** RNase-free workflow needs to be strictly followed during preparation and use of mRNA. Use a dedicated set of pipetters and filtered RNase-free pipette tips. Clean all gloves, tools and work surfaces with RNase decontamination solution. Use autoclaved RNase-free microtubes to store RNA solutions. Keep mRNA on ice at all times during injections and store at −80°C.
  a. Linearize pCS2+GCaMP6s with NotI.
    i. Linearize 10 μg of plasmid with NotI for 2h at 37°C.
      ***Note:*** Linearizing the plasmid ensures that the polymerase will fall off at the cut site, only transcribing the GCaMP6s sequence. NotI cuts after the SV40 polyA sequence in pCS2+, adding a polyA-tail to the GCaMP6s coding sequence for efficient translation in the embryo.
    ii. Run an aliquot of linearized plasmid on an agarose gel to verify linearization.
      ***Note:*** There should be a single linearized band and no circular plasmid.
    iii. Purify the linearized plasmid with GeneJet PCR Purification Kit or any other kit suitable for DNA purification. Follow the instructions of the kit.
      ***Note:*** Work should be RNase-free in this and all subsequent steps. It is advisable to use dedicated kits for RNase-free work to avoid contamination.
    iv. Elute DNA with 25 μl Elution Buffer (included with DNA purification kit) in a clean RNase free microtube.
    v. Measure DNA concentration with a spectrophotometer or Nanodrop.
    vi. Store linearized plasmid at −20°C. The linearized plasmid can be used for several RNA preparations.
  b. Transcribe capped mRNA for GCaMP6s using the SP6 mMessage mMachine Kit.
    i. Use 1 μg of linearized pCS2+GCaMP6s plasmid in a 20 μl reaction following the instruction manual of the SP6 mMessage mMachine kit.
    ii. Incubate the reaction for 2h at 37^o^C.
    iii. Digest the reaction with 1 μl DNase for 15 min at 37°C to get rid of the linearized plasmid.
    iv. Purify the mRNA with the GeneJet RNA clean up and concentration kit, following the instruction manual of the kit.
    v. Elute the purified mRNA with 15 μl RNase-free H_2_O in a clean RNase-free microtube.
    vi. Check an aliquot (0.5 μl) on an agarose gel to see that the prep is clean and not contaminated. You should see a sharp band.
      ***Note:*** A smear on the gel is a sign of RNA degradation. Degraded RNA is not suitable for injections.
    vii. Measure RNA concentration with spectrophotometer or Nanodrop.
      ***Note:*** Usually, one reaction will yield between 20–35 μg of mRNA.
    viii. Make 1 μL aliquots and freeze at −80°C.
      ***Note:*** mRNA can be stored frozen for up to 2 years.
      ***Note:*** For each new RNA construct, it is necessary to titrate the concentration of the RNA to find the optimal injection concentration. Perform blastomere injection with 2–3 different RNA concentrations and compare the results to find the concentration that yields the best expression level. In the case of GCaMP6s, a high expression level is generally desired as it usually gives a better signal-to-noise ratio, but excess expression can interfere with the dynamic range of Ca^2+^ signaling and may also lead to growth impairments and cytotoxicity.^21^^,^^22^
      ***Note:*** The same steps can be used to produce mRNA for any genetically encoded fluorescent protein. We frequently co-inject mCherry mRNA with GCaMP6s mRNA to facilitate animal screening, as mCherry is clearly visible under basal conditions by widefield epifluorescence even at relatively low expression levels.
4. For knockdown of GluN1 using morpholino injections, prepare antisense morpholino oligonucleotide.
  a. Reconstitute the MO in sterile water at 1 mM.
  b. Calculate working concentrations in mg/mL from the weight of the oligo on the data sheet.

***Note:*** Antisense morpholino oligonucleotide (MO) with a 3′ lissamine fluorescent tag, designed against the 5′ UTR of GluN1 (GluN1-MO) with the sequence CTGTGCCAAGCGGAGCCAATGTCCT is used to block GluN1 translation. A lissamine-tagged MO with a non-targeting sequence of CCTCTTACCTCAGTTACAATTTATA is used as a biologically inactive control (CTRL-MO).

***Note:*** For each new MO oligo, efficient working concentrations must be tested by titration. Perform blastomere injection with 2–3 different concentrations and check for efficient knockdown by either Western Blot or Immunohistochemistry with a specific antibody, in our case against GluN1 (see Kesner et al.^1^). Determine the lowest concentration needed for an efficient knockdown. Inject the same concentration of the CTRL-MO and make sure the effect that you see is specific to your MO oligo and not an unspecific effect due to injection artefacts or toxicity due to the MO oligo concentration. High MO concentrations may lead to developmental defects and higher mortality rates.

***Note:*** 1 mM MO solution should be stored at 21–25°C in a tightly sealed vial kept in a dark, humid environment (e.g. a sealed jar containing a small beaker of water).

### Calibrate injection pipettes

**Timing: 30 min**

For blastomere injection, a pressure microinjection system is used with calibrated pipettes to inject solution into embryos. We use a Tritech MINJ-D All-Digital Multi-Pressure microINJECTOR, which has a fill mode for front-loading pipettes. Blastomere injection pipettes need to be calibrated to dispense a precise volume (∼2 nL per injection for Xenopus eggs) to achieve ideal results while minimizing damage to the embryo.

**Figure 3:**
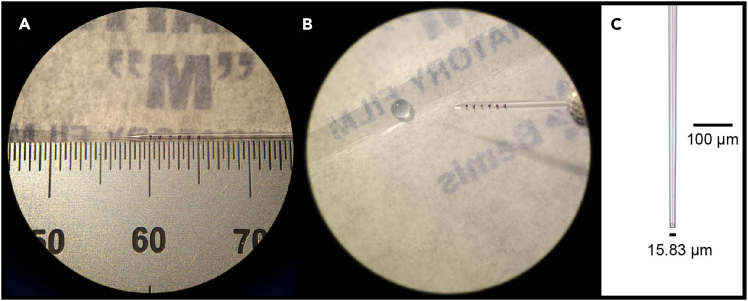
Marking and calibrating a glass pipette for blastomere injection (A) Draw 1 mm spaced marks on the barrel of the pipette with a fine-tip permanent marker to measure fluid volume. (B) Front load the pipette with RNase-free distilled water at least up to the 3 mm mark for calibration. (C) Closeup image of the tip of a calibrated pipette.

5. Pull Drummond microcaps pipettes on a Warner Flaming/Brown P-97 pipette puller to create injection micropipettes. Pull pipettes with tips that are approximately 1 cm long and can be broken back to 10–20 μm wide (Figure 3).

***Note:*** We use a 3 mm box filament with the following settings: P=225, Heat≈ramp, Pull=30, Vel=110, Time=200, which results in a one-step pull.

6. Place a piece of Parafilm with the lined face upwards to use as a working surface during the calibration process:
  a. Cut a small 5 cm square of Parafilm.
  b. Make a crease by folding it inwards towards the lined face.
  c. Detach the Parafilm from its lining.
  d. Place the Parafilm square under the dissecting scope, lined side facing up, on top of the lining paper or a small sheet of plain paper to reduce static.

***Note:*** Follow RNase-free workflow when calibrating pipettes: Use sterile gloves cleaned with RNase decontamination solution; liquid to be loaded into pipettes should be transferred using dedicated pipetters and filtered RNase-free pipette tips.

7. Place an injection pipette on the Parafilm.
8. Using a fine point permanent marker and ruler (decontaminated with RNaseZap and ethanol), make 1 mm graduation marks on the barrel of the pipette, which roughly corresponds to 100 nL of liquid (Figure 3A).

***Note:*** For Drummond 6.66 μl / 72 mm pipettes, the exact volume of 1 mm liquid within the barrel is 92.5 nL.

9. Set up the microinjection system and mount the pipette on the micromanipulator.
10. With the tip of the pipette in focus under a dissecting microscope, carefully break back the tip with Dumont #5 forceps about 0.5 mm from the tip.
11. Place a fresh sheet of creased Parafilm under the dissecting scope.
12. Pipette a drop of RNase-free water (1–5 μl) onto the crease.
13. Front load the pipette with the droplet of water, up to the 4 mm mark using the fill mode of the microinjector (Figure 3B).
14. Adjust the injection pressure and time to approximate 10 nL per injection (i.e., ∼10 injections to dispense 1 mm liquid in the pipette, between two marked lines).
  ***Note:*** An injection time of 1000 ms at an injection pressure of 15–25 psi is consistent with a suitable tip opening (Figure 3C).
  a. Keeping the tip of the pipette in the droplet of water, make 30 injections and observe the change in the fluid level inside the pipette.
  b. Adjust the injection pressure or time so that 30 injections dispense exactly 3 mm liquid.
    i. Increase injection pressure to dispense more liquid, or reduce injection pressure to dispense less liquid, repeating until the target of dispensing exactly 3 mm liquid with 30 injections is achieved.
    ii. If the pipette is not dispensing enough liquid under the maximum setting of 25 psi/1000 msec, break ∼0.5mm off the tip of the pipette with #5 forceps and try again.
    iii. If the pipette is dispensing too much liquid under the minimum setting of 15 psi/1000 msec, discard the pipette and start with a fresh one.
      ***Note:*** Do not use micropipettes that require a pressure below 15 psi: the tip opening will be too large, and you may damage the embryos.
  c. Record the calibrated injection pressure and time for the pipette.
15. Load a new pipette and repeat the calibration procedures until you have 4–5 calibrated pipettes.
16. Note the calibration settings for each pipette, and store them in a storage box with a foam insert with regularly spaced slots for inserting pipettes with their tips suspended to avoid damage.

***Note:*** For blastomere injections, the desired final injection volume of 2 nL can be achieved by reducing the injection time proportionately (i.e., For a pipette calibrated to dispense 10 nL per injection over 1000 ms, reduce the injection time to 200 ms to dispense 2 nL per injection).

## Key resources table

**Table:** 

REAGENT or RESOURCE	SOURCE	IDENTIFIER
**Antibodies**		

Mouse anti-GluN1 monoclonal antibody, 1:250 dilution	BD Biosciences	Cat# 556308; RRID: AB_396353
Goat anti-mouse Alexa fluor 488 antibody, 1:1000 dilution	Thermo Fisher	Cat# A-11029; RRID: AB_2534069

**Chemicals, peptides, and recombinant proteins**		

Tricaine (MS-222)	Sigma-Aldritch	Cat# A5040
L-cysteine hydrochloride	Sigma-Aldritch	Cat# C1276
Ficoll PM 400	Sigma-Aldritch	Cat# F4375
Sodium chloride	Fisher Scientific	Cat# S271
Potassium chloride	Sigma-Aldritch	Cat# P3911
Sodium bicarbonate	EMD	Cat# SX0320-1
Magnesium sulfate, heptahydrate (246.48)	EMD	Cat# MX0070-1
Calcium nitrate, tetrahydrate	Fisher Scientific	Cat# 13477-34-4
Calcium chloride, dihydrate	Sigma-Aldritch	Cat# C3881
HEPES free acid	VWR	Cat# CA-EM5320
Sodium hydroxide	Fisher Scientific	Cat# S318
UtraPure RNase-free distilled water	Invitrogen	Cat# 10977-015
Human chorionic gonadotropin (hCG), 10000 IU	Sigma-Aldritch	Cat# CG-10
Pregnant mare serum gonadotropin (PMSG), 1000 IU	Prospec Bio	Cat# HOR-272
0.9% sodium chloride injection USP, 10 mL vial	Pfizer	NDC: 00409-4888-10
NotI	NEB	Cat# R3189S
UltraPure Agarose	Life Technologies	Cat# 16500-100

**Deposited data**		

Injection dish 3D print file	–	Github: https://github.com/RuthazerLab/Hemimosaic-3d-models

**Experimental models: Organisms/strains**		

Albino *Xenopus laevis* (male and female)	Xenopus 1	Cat# 5215; RRID: XEP_Xla300
ElasGFP:Tubb2-GCaMP6s transgenic *Xenopus laevis* (frozen sperm)	NXR	Cat# NXR_0107

**Oligonucleotides**		

GluN1-MO 3′lissamine	Gene Tools	–
CTRL-MO 3′lissamine	Gene Tools	–

**Recombinant DNA**		

pCS2+ GCaMP6s	Cut from pGP-CMV-GCaMP6s #40753 from Addgene	Plasmid# 40753, RRID: Addgene_40753
pCS2+ jRGECO1a	Cut from pGP-CMV-NES-jRGECO1a #61563 from Addgene	Plasmid# 61563, RRID: Addgene_61563

**Software and algorithms**		

Suite2p 0.14.3	Pachitariu et al.^23^	RRID: SCR_016434; Github: https://github.com/MouseLand/suite2p

**Other**		

All-Digital Multi-Pressure microINJECTOR (or other microinjection system capable of frontloading pipettes) with foot pedal	Tritech	Cat# MINJ-D
Compressed nitrogen gas	Linde	Cat# Ni 4.8K
Singer “Pantographic” Micromanipulator, MK-1	Singer Instruments	Cat# MK-1
P-97 flaming/brown micropipette puller	Sutter Instrument	Cat# P-97; RRID: SCR_016842
3 mm Box Filament	Harvard Apparatus	Cat# FB330B
Drummond MicroCaps (6.66 μl, 72 mm)	VWR	Cat# 53507-146
Dumont #5 forceps	FST	Cat# 11252-20
GeneJet PCR purification kit	Life Technologies	Cat# K0702
GeneJet RNA clean up and concentration kit	Life Technologies	Cat# K0841
SP6 mMessage mMachine Kit Ambion	Thermo Fisher	Cat# AM1340; RRID: SCR_008452
RNaseZap™ RNase decontamination solution	Thermo Fisher	Cat# AM9780
10 μl filtered pipette tips	Ultident	Cat# 4117NSF
UVette photometer	VWR	Cat# CA89094-396
6-well plates	Fisher Scientific	Cat# 087721B
Sterile petri dishes (60 mm, 100 mm diameter)	Thermo Fisher	Cat# FB0875713A, FB0875713
Eye dressing forceps	FST	Cat# 11150-10
Razor blade	Acuforge	Cat# AGBL-7033-0000
Iris scissors	WPI	Cat# 501758
Plastic transfer pipettes	Fisher Scientific	Cat# 137177M
Disposable underpads (17×24 in)	VWR	Cat# 56617-014
Bone shears	FST	Cat# 16150-24
0.5 mL disposable insulin syringe (28G)	Fisher Scientific	Cat# 1482679
Parafilm (4″×250′)	VWR	Cat# 52858-032
#184 Sylgard Kit	Paisley	Cat# AVDC0184

## Materials and equipment

**Table:** Modified Barth’s solution (MBSH) – 10× concentrated stock

Reagent	Final concentration	Amount
NaCl	88 mM	51.4 g
KCl	1 mM	0.75 g
NaHCO_3_	2.4 mM	2.0 g
MgSO_4_ x7H_2_O	0.82 mM	2.0 g
Ca(NO_3_)_2_ x4H_2_O	0.33 mM	0.78 g
CaCl_2_ x2H_2_O	0.41 mM	0.6 g
HEPES free acid	10 mM	23.8 g
NaOH	N/A	N/A
ddH_2_O	N/A	N/A
**Total**	**N/A**	**1 L**

Adjust pH to 7.4 with NaOH. Store at 4°C indefinitely.

### MBSH—0.1× working solution (animal rearing medium)

Dilute 100 mL of 10× MBSH concentrated stock in 9.9 L ddH_2_O to make 10 L working solution.

Store at 21–25°C indefinitely.

### MBSH—1× working solution (testis storage medium)

Dilute 10 mL of 10× MBSH concentrated stock in 90mL ddH_2_O to make 100 mL working solution.

Store at 4°C indefinitely.

### 0.2% MS-222 in 0.1× MBSH (tadpole euthanasia and frog anesthesia solution)

Add 1 g MS-222 powder to 500 mL 0.1× MBSH and adjust pH to 7.4.

Store at 4°C for up to 4 months. Protect from light.

***Note:*** MS-222 overexposure may cause skin, eye and respiratory irritation. When handling MS-222 powder, wear a lab coat and nitrile gloves and work in an area free from drafts. MS-222 waste solution may need to be collected and disposed of as hazardous chemical waste.

### 0.02% MS-222 in 0.1× MBSH (tadpole anesthesia solution)

Dilute 50mL of 0.2% MS-222 in 450 mL 0.1× MBSH to make 500 mL working solution.

Store at 4°C for up to 4 months. Protect from light.

### 2% Ficoll in 1× MBSH (blastomere injection medium)

Dissolve 2 g Ficoll powder in 100 mL 1× MBSH. Use low heat and stirring to facilitate dissolving.

Store at 4°C or −20°C for up to 4 months.

***Note:*** Before using stored Ficoll solution from 4°C, always check if there is solid deposit or biofilm contamination. If the solution is contaminated, discard and make new solution. Alternatively, Ficoll solution can be stored at −20°C to prevent biofilm formation.

### 1% Ficoll in 0.1× MBSH (embryo incubation medium)

Dissolve 1 g Ficoll powder in 100 mL 0.1× MBSH.

Store at 4°C for up to 4 months.

### Pregnant mare serum gonadotropin, 1,000 IU/mL

Add 1 mL 0.9% saline to 1000 IU vial.

Store at 4°C for up to 3 months.

### Human chorionic gonadotropin, 1,000 IU/mL

Extract 1 mL from a fresh 10 mL vial of 0.9% sterile saline and use to suspend 10000 IU hCG. Transfer hCG solution back into the original 10 mL vial of 0.9% saline to achieve 1000 IU/mL.

Store at 4°C for up to 3 months.

## Step-by-step method details

### Prime female frogs for egg collection

**Timing: 3–4 days**

This section describes steps to induce ovulation in female frogs. As eggs for *in vitro* fertilization and blastomere injection must be collected fresh, all timings are provided relative to the time of planned blastomere injection.

**Figure 4:**
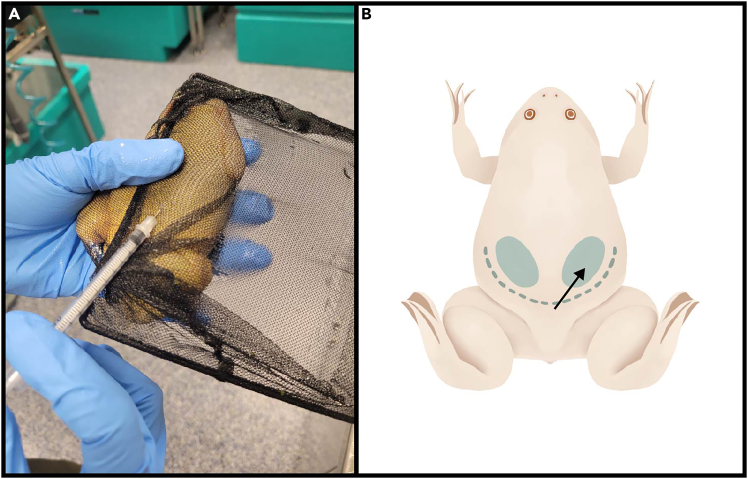
Priming injection for female frog (A) Restrain the frog with an aquarium fish net, and deliver priming hormone through subcutaneous injection into the dorsal lymph sac. (B) Diagram of injection locations in the dorsal side of the frog. Dotted blue line shows the approximate caudal limit of the dorsal lymph sac. Blue shaded areas are ideal locations for injections. Insert the syringe needle at a shallow angle, in the direction indicated by the black arrow.

1. 3–4 days before planned blastomere injection: Prime adult female frog with 50 IU PMSG by subcutaneous injection into the dorsal lymph sac (Figure 4).
  a. Load 0.5 mL insulin syringe with 50 μl of 1000 IU/mL PMSG.
  b. Restrain the female frog by placing it in an aquarium fish net.
  c. Fold the net over the frog and hold the frog firmly in your non-dominant hand so that the frog is not moving and the dorsum of the frog is easily accessible for injections.
  d. Insert the syringe needle subcutaneously into the posterior medial region of the dorsal side of the frog.
  e. Once the needle penetrates the skin and enters the lymph sac, slowly inject the hormone.
    ***Note:*** Insert the needle at a shallow angle to avoid penetrating through the dorsal lymph sac into the muscle underneath.
  f. Remove the syringe and place the primed frog in a separate tank.
2. 16–20 h before planned blastomere injection: Inject primed female frog with 400 IU hCG.
  a. Prepare a temporary holding tank for the frog (e.g., a plastic rat tank with an air-permeable cover). Fill it to ¾ with system water.
  b. Fill a 0.5 mL insulin syringe with 0.4 mL of 1000 IU/mL hCG.
  c. Inject the hormone subcutaneously into the dorsal lymph sac as described in Step 1.
  d. Place the injected frog into the temporary holding tank.
  e. Cover the tank with a cloth sheet to reduce disturbances.
  f. Keep the frog in the temporary holding tank until the time for egg collection.

***Note:*** Do not feed the frogs while they are in the temporary holding tank, as solid waste will cause rapid decline in water quality.

***Note:*** The PMSG priming step promotes oocyte maturation, but only the hCG injection step is necessary to induce ovulation. If there is not enough advance time to perform PMSG priming, frogs can be made to spawn with just the hCG injection.

***Note:*** To increase the probability of obtaining eggs, it is recommended to prime 2 female frogs, as some females do not spawn even after completing all priming procedures.

3. On the day of planned blastomere injection: Check if the primed female frogs are ovulating.

***Note:*** Noticeable signs for ovulation include a red to purple cloaca and eggs present in the water or emerging from the cloaca. Troubleshooting 1.

***Note:*** Frogs injected with hCG in the afternoon or evening of the previous day will typically start ovulating in the morning. Eggs released into the water will cause the water quality to quickly deteriorate, therefore it is important to monitor water quality and switch out dirty water in the holding tank if spawning frogs are not immediately used for egg collection.

**CRITICAL:** Ensure at least one primed frog is producing eggs before proceeding with testis harvesting from male frogs in the next step.

### Extract testes from a male frog

**Timing: 1 h**

This step details the harvesting and storage of testes from a male frog for *in vitro* fertilization.

***Note:*** To avoid unnecessary sacrificing of a male frog and to use the testes at their maximum viability, testis harvesting should only be performed when you have eggs available to proceed with *in vitro* fertilization.

4. Anesthetize an adult male frog by immersing in 0.2% MS-222.
  a. Place the male frog in a small (∼1 L) tank and fill it with ∼500 mL cold 0.2% MS-222 so that the frog is submerged.
  b. Wait 20–30 min for the animal to be fully anesthetized.
  c. Test for withdrawal reflexes by pinching the frog in its thigh or webbing of the foot.
  d. When no reflexes are present, move the frog onto a disposable soaker pad to perform dissection.

***Note:*** The 0.2% MS-222 solution used for male frog anesthesia can be reused up to 10 times within its shelf life. After removing the frog from the anesthesia tank, clean out any debris from the MS-222 solution and carefully pour it back into a glass bottle for storage and reuse.

5. Fill three 60 mm petri dishes with 1× MBSH, and chill them on ice until use.

***Note:*** The cold 1× MBSH will be used for rinsing and storing testes. The high salt concentration renders the sperm immobile.

**Figure 5:**
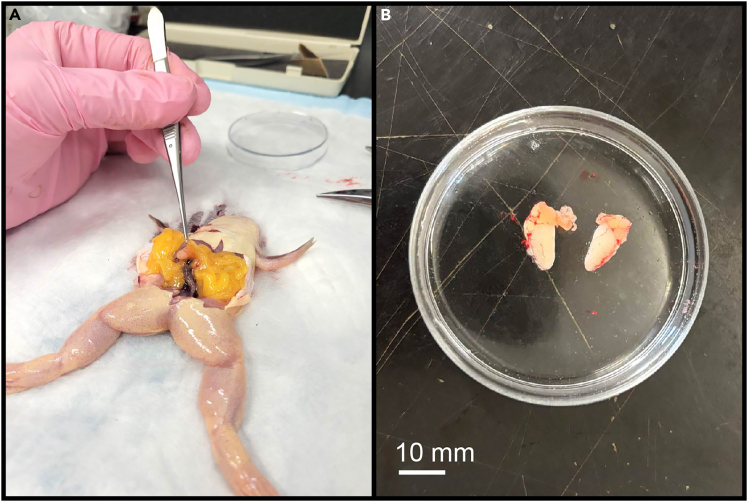
Extraction of testes from male frog (A) Testes can be found attached to fat tissue located in the abdomen of the frog. (B) Once a testis is located, carefully cut it out with a piece of fat tissue attached, then rinse and store the extracted testis in chilled 1× MBSH.

6. Place the frog belly-up on the soaker pad.
7. Use sharp shears to decapitate the frog. Ensure the spinal cord is completely severed.
8. Use forceps to pick up the skin on the belly of the frog.
  a. Using a razor blade or dissecting scissors, make one incision along the midline of the frog, from belly to sternum.
  b. Make perpendicular incisions at both ends of the initial incision.
  c. Cut through the skin and abdominal wall.
  d. Fold both back to expose the abdominal cavity.
9. Locate the testes by pulling out the fat tissue in the abdominal cavity (Figure 5).

***Note:*** The fat tissue is yellow in color and located on both lateral sides of the frog, below the level of the liver. The testes are kidney-shaped structures around 5–10 mm in length and white to pale yellow in color, and will be attached to the fat tissue.

10. Using forceps, grip the fat tissue next to a testis and cut out the testis with a small piece of fat tissue attached. Repeat for the other testis.

***Note:*** Always handle the testes by the attached fat tissue to avoid damaging them.

11. Rinse the extracted testes in one of the small petri dishes with chilled 1× MBSH, then transfer each testis into a separate small petri dish with cold 1× MBSH. Store the testes at 4°C until use.

***Note:*** Fresh, whole testes can be kept in 1× MBSH at 4°C for up to 2 weeks from the time of harvest. Change the testes into fresh 1× MBSH after 1 week. Each testis can be cut into smaller pieces and used to fertilize several batches of eggs, but cut testes can only be stored and reused for up to 1 week, as the quality of sperm will deteriorate quickly once the testis is cut.

12. Wrap up the carcass of the male frog with the soaker pad and place at −20°C.
13. Once the carcass is frozen, dispose of it appropriately.

### Egg collection from a female frog

**Timing: 15 min**

This step describes a way to manually restrain and “squeeze” a spawning female frog to collect eggs into a petri dish. Wear a lab coat and nitrile gloves, and work over an open, waterproof counter, free of sharp objects that may injure the frog in case it escapes.

***Note:*** Only fresh eggs collected as soon as they are laid can be used for *in vitro* fertilization. Spawning females may have already released eggs into the holding tank water before the time of egg collection, but these eggs will not be of adequate quality and should be discarded.

**Figure 6:**
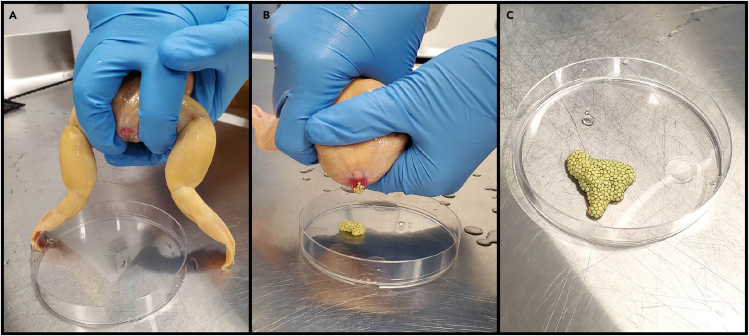
Restraining and squeezing a female frog to collect eggs for in vitro fertilization (A) Hold the frog with your dominant hand, using your other hand to help support the frog’s belly. Spread the frog’s hind legs with your index and middle finger to expose the cloaca, and massage the frog’s back and belly from the sacrum towards the cloaca to facilitate egg release. (B) Alternatively, you can also spread the frog’s hind legs using your index fingers from both hands, which will give better access to the frog’s back for massaging. (C) Healthy eggs should naturally form clumps and stick to the bottom of the Petri dish, each egg surrounded in jelly coat and well separated from each other.

14. Prepare a sterile 100 mm petri dish for collecting eggs.
15. Place the holding tank containing the spawning female frog in a location where it will be easy to transport the animal between the tank and the counter.
16. Restrain the female frog in a secure hold with both hands (Figure 6A).
  a. Using your dominant hand, secure the female frog so that the dorsal side of the frog is resting against the palm and the head of the frog is facing the wrist, with the base of your palm covering the frog’s eyes.
  b. Place your index and middle finger between the two hind limbs of the frog, and wrap your thumb and 4^th^ and 5^th^ finger around the trunk.
  c. Once the frog is firmly gripped in your dominant hand, gently lift it out of the water and use your other hand to cover and support the ventral side of the animal, with the belly resting against the palm and head facing the wrist.
  d. Transport the frog to the counter in this position.

***Note:*** The frog may struggle when held. Covering the eyes and applying some pressure to your hold should help make the frog settle down. Take care not to apply too much pressure that will injure the frog. Make sure your grasp is secure enough that the animal will not slip loose and escape before attempting to transport it.

17. “Squeeze” the frog to collect eggs (Figures 6A and 6B, Methods video S1).
  a. Position the frog’s cloaca over the center of the petri dish.
  b. Using the two fingers of your dominant hand placed between the frog’s legs, gently spread the legs apart to expose the cloaca.
  c. Apply gentle pressure to the frog’s trunk with your thumb and 4^th^ and 5^th^ fingers, and use your thumb from your other hand to massage the frog’s back from the sacrum towards the cloaca to facilitate egg release.
  d. Collect the eggs as they are released into the petri dish.

***Note:*** If no eggs come out or the flow of egg release is slow, try massaging different parts of the frog’s back and belly, or repeatedly releasing and reapplying pressure. There is often a “sweet spot” or a specific massaging pattern that will give a good flow. Be careful when applying pressure to not injure, bruise or create excessive stress. If you observe excessive mucus developing on the skin (a sign of stress), stop squeezing and return the animal to the holding tank.

***Note:*** Healthy eggs form clumps, each egg surrounded by a jelly coat and well separated from each other, firm and spherical in shape, pale green or pale yellow in color (Figure 6C). They should be released out of the cloaca with relatively little accompanying fluid, and stick to the bottom of the petri dish. If the eggs are deformed, tubular, or merging into a mush, they are likely of poor quality. Troubleshooting 2.

**CRITICAL:** Keep the petri dish with the eggs dry – do not leave the eggs immersed in system water, as it will cause egg viability to decline. If water dripped into the dish during egg collection, decant the water or dab it off with a Kimwipe, taking care not to touch the eggs. Use the collected eggs for *in vitro* fertilization within 30 min, before the eggs dry out.

18. Return the female frog to its tank.

***Note:*** Keep a record of when frogs have been used for egg collection. Female frogs in a laboratory colony should be cycled so that each frog has 2–3 months of rest between uses.

### *In vitro* fertilization

**Timing: 1.5 h**

This step details *in vitro* fertilization using the testes and eggs harvested from the previous steps. Prepare a stopwatch or timer to keep track of passing time, as the process from the start of *in vitro* fertilization to the end of blastomere injection will be time sensitive.

19. Prepare the equipment and solutions for *in vitro* fertilization.

***Note:*** Have at least 2 L each of 0.1× MBSH and double distilled water available in carboys or other containers that allow easy dispensing.

**Figure 7:**
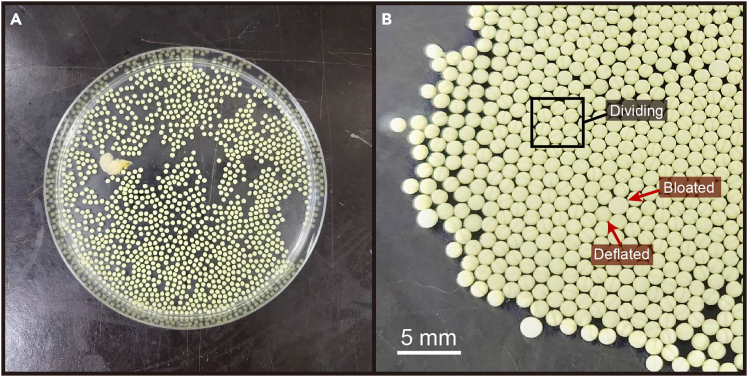
In vitro fertilization (A) After mincing a piece of testis and mixing it with the eggs, adding 0.1× MBSH will activate the sperm and initiate fertilization. Eggs are spread out into a single layer at the bottom of the dish. (B) At 22°C ambient temperature, the first cell division will start at 75–90 min post-fertilization. Healthy eggs will start to form cleavage, while unhealthy or unfertilized eggs will not divide, and will appear bloated, deflated or start to disintegrate.

20. Take a testis out of 4°C storage and transfer it into the petri dish with the eggs.
  a. In the petri dish with the eggs, find an empty space and place a 50 μl drop of 1× MBSH.
  b. Using forceps, transfer the testis into the drop of 1× MBSH.
  c. Cut off a piece of testis with a clean razor blade.
    ***Note:*** The amount of testis to use will depend on the number of eggs in the clutch – around 1/8 of a 10 mm long testis will fertilize ∼300 eggs.
  d. Return the unused portion of the testis to its dish of 1× MBSH and put it back in 4°C storage.
  e. Using the razor blade, mince the extracted piece of testis into a fine paste.
  f. Use a 200 μl pipette to distribute macerated testis over the eggs.
  g. Use the tip of the pipette to gently mix the eggs with the testis and move some eggs over the area where the testis was minced.
    ***Note:*** You can also cut a piece of testis from its dish, crush it in an Eppendorf tube filled with 0.1× MBSH using scissors and then a mortar, and flood the eggs with this mixture.
    ***Note:*** The eggs are protected by a jelly coat and will not be easily damaged by the pipet tip, but be gentle nonetheless!
  h. Add 500–800 μl of 0.1× MBSH to the eggs, just enough to cover the eggs in a thin film of liquid.
  i. Mix the eggs with the pipette tip and spread them out into a single layer.
    ***Note:*** The 0.1× MBSH will activate the sperm and allow it to move and fertilize the eggs. The small buffer volume restricts the sperm to the area around the eggs (Figure 7A).
    **CRITICAL:** The addition of 0.1× MBSH marks time point zero of the fertilization. Start a timer to monitor your progress.
21. After the initial addition of 0.1× MBSH, leave the eggs for 5 min to allow fertilization.
22. At time point t = 5 min, fill the petri dish with 0.1× MBSH to fully immerse the eggs in buffer. Let stand for another 5 min.
23. During the waiting time, prepare ∼50 mL of 2% w/v L-cysteine in ddH_2_O. Adjust pH to 8.0 with 5M NaOH.

***Note:*** L-cysteine is an irritant. If the powder or solution come in contact with your skin, thoroughly wash the affected area with water.

24. At time point t = 10 min, carefully decant the MBSH from the dish into a clean small beaker.

***Note:*** The eggs will usually stick to the bottom of the dish. Pick up any eggs that fall into the beaker with a plastic transfer pipette and return them to the dish.

25. Fill the dish with the 2% cysteine solution. Incubate for 7–10 min, swirling the plate gently from time to time.

***Note:*** Cysteine treatment separates the eggs from their jelly coat, making them accessible for blastomere injection. Do not leave the eggs in cysteine solution for more than 10 min as it might damage the eggs.

26. Once the eggs are fully separated from their jelly coat, carefully decant the cysteine solution.
27. Pour the eggs into a 250 mL beaker filled with 100 mL of ddH_2_O.

***Note:*** Healthy, fertilized eggs will sink to the bottom of the beaker. Remove and dispose of any bloated or floating eggs.

28. Decant the water and wash the eggs two more times with ddH_2_O, and three more times with 0.1× MBSH.
29. If the liquid still turns cloudy in the last wash, continue to wash with 0.1× MBSH until the wash comes off clear.

***Note:*** When adding liquid to the beaker with eggs for washing, let the liquid run along the side of the beaker in a slow flow. Avoid agitating the eggs, as too much agitation may lead to developmental defects.

30. Decant the final wash and pour the eggs into a clean 100 mm petri dish filled with 0.1× MBSH.
31. Check the eggs under a dissecting scope and remove any remaining unhealthy (bloated) or damaged eggs.

**CRITICAL:** To ensure a healthy batch of animals, it is imperative to rigorously clean the embryos, as discharge from unhealthy eggs will negatively affect healthy ones. Healthy, fertilized eggs should appear solid and compact, round with a slightly flattened animal pole.

32. At 22°C ambient temperature, the first cell division will start about 75–90 min post-fertilization.

***Note:*** If there is a need to slow down the start of first cell division, keep the eggs at a lower temperature (14–18°C) after cleaning them.

***Note:*** After the embryos start dividing, you can start to separate dividing embryos from non-dividing ones. Embryos that appear bloated or deflated will not be viable and can be discarded (Figure 7B).

### Blastomere injection of mRNA or morpholino

**Timing: 30 min**

The following section describes the process of blastomere injection and handling of embryos following the injection.

**Figure 8:**
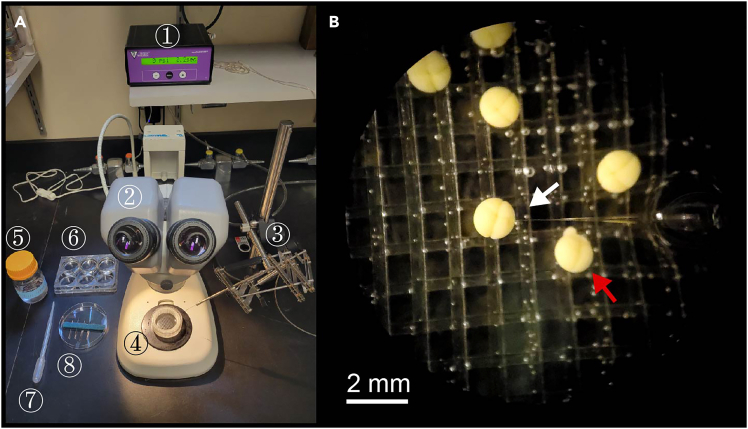
Blastomere injection (A) Equipment setup for blastomere injection. ①Microinjector ②Dissecting scope ③Pantographic micromanipulator ④Injection chamber ⑤Blastomere injection medium (2% Ficoll in 1× MBSH) ⑥6-well plate to store injected embryos ⑦Transfer pipette to move embryos ⑧Calibrated injection pipettes. (B) Close-up of injection pipette in position for blastomere injection into a 4-cell stage embryo (white arrow). A shallow dent can be seen forming on the surface of the blastomere where the pipette tip is pressing down. The dent will disappear once the pipette tip penetrates the cell membrane. Note that the embryo indicated with a red arrow has been damaged and contents can be seen leaking out. This embryo should be discarded.

33. While waiting for the first cell division, set up the blastomere injection station and prepare relevant equipment and solutions (Figure 8A).
  a. Prepare a clean 6-well plate to keep injected eggs.
  b. Fill wells with 2% Ficoll/1× MBSH solution.
    ***Note:*** The number of wells to use depends on the number of embryos to be injected (about 30–60 embryos per well).
  c. Prepare your injection solution, diluted to the appropriate concentration.
    i. For GCaMP6s mRNA, use RNase-free ddH_2_O to dilute to a working concentration of 0.5 μg/μl.
      ***Note:*** Keep the diluted working solution on ice. The working solution can be reused 2–3 times. Freeze the solution at −80°C after injections.
    ii. For MO, dilute your MO with ddH_2_O to 5 ng/nL.
      ***Note:*** Spin MO for 1 min at 16.1 × *g* to reduce needle clogging. Diluted MO can be kept at 21–25°C during injections and at 4°C for longer term storage.
      ***Note:*** The injection concentrations listed above are recommended values. It may be necessary to test several different mRNA or MO concentrations to find one that produces the best expression for you.
      **CRITICAL:** If working with mRNA, follow RNase-free workflow as described in the pipette calibration step.
  d. Place the blastomere injection chamber inside a 60 mm petri dish.
  e. Fill the dish with 2% Ficoll/1× MBSH.
    ***Note:*** Ficoll makes the solution viscous and helps stabilize the embryos in the dish during injection. The high salt concentration shrinks the embryos and prevents leakage of intracellular material from the embryo after withdrawal of the injection pipette. Let the Ficoll solution warm to 21–25°C before use.
  f. Mount a calibrated injection pipette onto the micromanipulator and connect it to the microinjection system.
  g. Verify that the calibration is still accurate by filling the pipette with RNase-free water and performing test injections as in the calibration step.
  h. Set the microinjection system to a slightly positive balance pressure (4 kPa) for injections.
34. When embryos reach the end of 2-cell stage, start blastomere injection.
  ***Note:*** At 22°C ambient temperature, embryos typically enter 2-cell stage at t = 75–90 min post-fertilization, with each stage lasting 20–30 min. For best results (good hemimosaicism), start injections towards the end of the 2-cell stage. If embryos start dividing at t = 90 min, start injections around t = 105–110 min. This ensures that most of the embryos have almost completed their first cell division and reduces leakage of mRNA into the other blastomere. In general, embryos can be injected from 2-cell stage through 4-cell stage to produce hemimosaicism, giving a window of ∼30 min for injections. With practice, it is possible to inject up to 300 embryos within this time.
  ***Note:*** If injecting at the 4-cell stage, also inject 1 blastomere only, preferably in one of the dorsal blastomeres. The dorsal blastomeres are the smaller two of the four blastomeres, although in some batches the sizes of the dorsal and ventral blastomeres may be similar.
  a. Select 30–100 healthy dividing embryos and transfer them to the injection chamber.
    i. Place the eggs in a single layer in the center of the chamber.
    ii. Let the embryos fall into rows on the mesh grid and ensure the embryos are all in areas that can be reached by the injection pipette (not too close to the rim of the dish).
      ***Note:*** Placing the embryos in the injection dish before loading your pipette gives the embryos time to adjust in the high salt buffer to minimize leakage.
  b. Pipette ∼1 μl injection solution onto a sheet of parafilm, prepared as in the calibration step.
  c. Front load the injection pipette with injection solution.
    ***Note:*** The fill volume will depend on the number of embryos to be injected. Calculate the volume needed and load the pipette accordingly, adding a bit more to ensure you don’t run out during injections (the positive balance pressure will make the pipette leak out small volume of solution during injections). For 200 embryos and an injection volume of 2 nL per embryo, fill the pipette a bit above the 4 mm graduation mark (one graduation mark corresponds to 50 injections). For MO, we inject about 10 ng into one blastomere at the two-cell stage of *in vitro* fertilized embryos to create bilateral hemimorphant animals.
  d. Place the injection dish and injection pipette in position for injections.
    i. Place the injection dish under the dissecting scope.
    ii. With your dominant hand, adjust the micromanipulator so that the injection pipette is at a 45° angle from horizontal, and the tip of the pipette can easily reach the bottom of the injection chamber and easily maneuver over a large area within the chamber.
    iii. Use your other hand to steady and move the injection dish.
      ***Note:*** We use a Singer MK1 pantograph for rapid positioning of the pipette tip, but it is also possible to mount the pipette in a 3-dimensional coarse micromanipulator such as a Narishige M152.
  e. Perform blastomere injection.
    i. Focus the dissecting scope on the embryo to be injected.
    ii. Insert the injection pipette just below the surface of one of the blastomeres, inject once, then pull out the pipette. (Figure 8B).
    iii. Proceed to the next embryo and repeat.
      **CRITICAL:** Check from time to time (every 30–50 injections) that the meniscus in the pipette is descending and the pipette is not clogged.
      **CRITICAL:** Use the mesh grid to keep track of the embryos as you inject them. Start at one corner of the grid and work along the rows. Most of the time, you will not be able to tell whether an embryo has been injected based on its appearance, so it is crucial to use the grid as a visual aid. You can carefully move the dish translationally to access different parts of the chamber for injections if the micromanipulator does not have enough range to reach all areas in the dish. Avoid rotating the chamber as it may cause you to lose track of your progress.
  f. After completing injections for all embryos in the dish, move the injection pipette away from the dish, taking care not to damage the tip.
  g. Transfer the embryos into the 2% Ficoll solution in the prepared 6-well plate.
  h. Transfer the next batch of 30–100 embryos into the injection dish and repeat the injection process.
    ***Note:*** Between batches, check that the injection pipette is not clogged or broken and that there is enough solution left in the pipette for the next batch. Troubleshooting 3.
35. Once all injections are completed, place the 6-well plate with the injected embryos into an 18°C incubator.
36. After 2–3 h, transfer the embryos into 1% Ficoll/0.1× MBSH and keep at 18°C for 12–24 h.

***Note:*** At this step, keep a maximum of 30 animals per well to avoid overcrowding.

37. After 12–24 h, transfer the embryos into animal bowls filled with 0.1× MBSH.

***Note:*** Ensure the embryos are given sufficient space – approximately 50 embryos per 250 mL medium.

***Note:*** Embryos may start leaking once transferred out of the Ficoll solution. Minor leakage of cell content is normal, the embryos will still develop normally. Clean out any resulting debris, as well as any embryos that become bloated or deflated.

38. Keep embryos in an incubator set to an appropriate temperature (between 16^o^C and 25^o^C) for development.

***Note:*** The temperature to keep the embryos at should be chosen based on the desired rate of growth – embryos will develop faster at higher temperatures.

39. Monitor the embryos for the following days to make sure they are developing normally.

***Note:*** Replace the rearing solution whenever it becomes dirty. Remove any embryos that fail to develop, as these will eventually die and promote bacterial growth.

***Note:*** The bulk of embryo mortality occurs in the first 2–3 days post-fertilization, and the embryos will need to be closely monitored and cleaned during this period to prevent dead embryos from affecting healthy ones. After animals reach stage 22 (at which point their bodies will grow long and unfurl into the shape of rice grains) they are likely to survive and can be checked less frequently.

40. Allow the animals to grow to the desired stage for your experiment.

### Screening animals for hemimosaicism (GCaMP6s mRNA)

**Timing: 1–2 h per screen**

The following section describes how to perform two screens on animals injected with GCaMP6s mRNA to identify individuals with good hemimosaic fluorescent protein expression. Animals injected with lissamine-tagged morpholino can be screened with the same procedures, screening for red lissamine fluorescence instead of green GCaMP fluorescence. The first rough screen at an earlier developmental stage is optional, recommended for more efficient processing of larger batches of animals.

**Figure 9:**
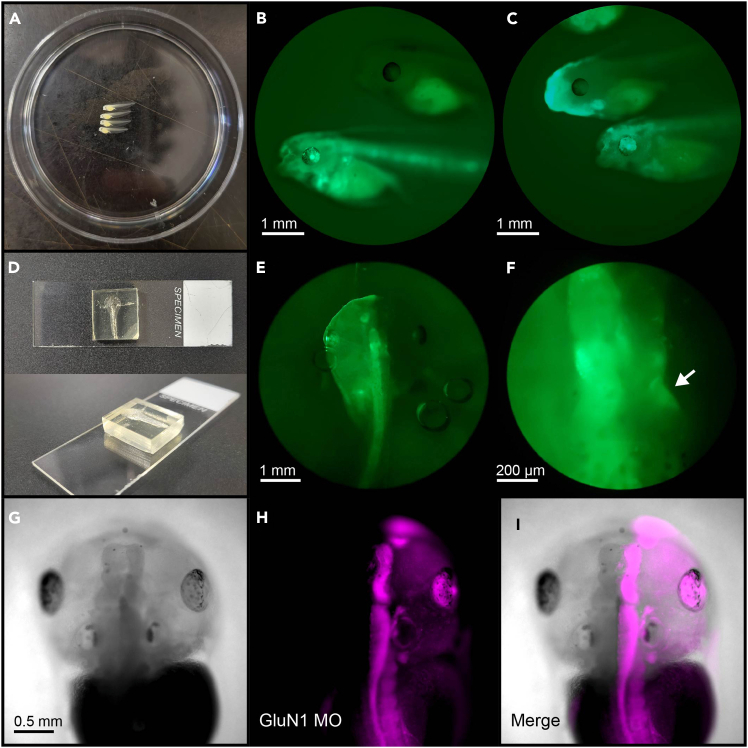
Screening animals for hemimosaicism (A) Stage 43 tadpoles anesthetized and lined up in a 60 mm Petri dish for screening. (B) GCaMP6s fluorescence in two stage 43 tadpoles. Bottom tadpole is an example of a “good” animal, showing bright fluorescence in both the eye and spinal cord. Top tadpole does not show noticeable fluorescence in any parts other than the stomach. The top tadpole would be safe to discard if the other side of the animal does not show good fluorescence as well. (C) Examples of GCaMP6s fluorescence in two more stage 43 tadpoles. Bottom tadpole shows good fluorescence in the eye and brain. In comparison, although parts of the head and the heart of the top tadpole are fluorescent, there is no noticeable fluorescence present in the central nervous system, making it less likely to be a good hemimosaic animal. (D) Example of a custom screening chamber for stage 44 and older animals. The impression of a tadpole is carved into a block of Sylgard elastomer. The shape and size of the impression should be adjusted to snugly fit a tadpole and a drop of medium and allow a cover slip to be placed over the tadpole without compressing it. (E) GCaMP6s fluorescence in a stage 48 tadpole, showing bilateral hemimosaic distribution of fluorescent protein. The left half of the animal shows bright green fluorescence in most regions, whereas the right half is only fluorescent in parts of the tectum. (F) Close-up image of tadpole in (E), focusing on the tectum. GCaMP fluorescence can be seen in the entire left tectal hemisphere, as well as the neuropil region of the right tectal hemisphere (white arrow). (G–I) Widefield images of a stage 45 GluN1 MO tadpole, adapted from Kesner et al.^1^ (G) Brightfield, (H) MO-lissamine fluorescence, (I) merged image.

41. Stage animals according to Nieuwkoop & Faber.^24^ When animals reach Stage 37–43 (animals appear long and flat and naturally lay on their side inside a petri dish), perform a first rough screen for candidate animals with hemimosaicism. (Figure 9A).
  a. Fill a small petri dish with 0.02% MS-222 in 0.1× MBSH for tadpole anesthesia.
  b. Add ∼10 tadpoles to the dish and wait until they stop moving.
  c. Place the dish under a widefield screening scope equipped with a fluorescence lamp.
  d. With a fine paintbrush, line up 3–4 tadpoles in the center of the dish so that their backs are facing the same direction.
  e. Check and note green fluorescence levels for each animal.
    ***Note:*** Animals with good expression of GCaMP6s will usually display bright fluorescence in the eyes.
  f. Carefully turn the tadpoles over by gently pushing their spine with the paintbrush. Check fluorescence on the other side of the animals.
  g. Keep animals with bright fluorescence on one side. Discard animals with low or no fluorescence (Figures 9B and 9C).
  h. Repeat the process until all animals have been screened.
  i. Keep the animals with bright and correctly distributed fluorescence in a 6-well plate or small animal bowl filled with 0.01× MBSH until the next screen.
42. The second, finer screen can be performed on animals at Stage 44 or later (head and tail of animals fully defined, animals can lay on their belly).
  a. Anesthetize an animal by placing it in a small petri dish with 0.02% MS-222.
  b. Place the animal on its belly in a droplet of 0.02% MS-222 in a large petri dish.
    ***Note:*** Alternatively, a custom screening chamber can be made by carving the shape of a tadpole out of a 2 × 2 × 0.5 cm piece of Sylgard silicone elastomer and placing it on a glass slide (Figure 9D).
  c. Under the screening microscope, check the animal for green fluorescence. Identify individuals with hemimosaic expression (Figures 9E and 9F).
    ***Note:*** Ideal hemimosaic animals should have bright fluorescence in the expressing lateral half and no fluorescence on the contralateral half, except for the neuropil region of the tectum (retinal ganglion cell axon terminals that crossed over from the expressing side), as well as the gut and kidneys.
    ***Note:*** The basal fluorescence of GCaMP6s under widefield epifluorescence is modest and diffuse, which can make screening difficult. Co-expressing GCaMP with another brighter fluorophore (by injecting a mixture of mRNA for both fluorophores) can facilitate the screening process. Troubleshooting 4.
    ***Note:*** The completeness of hemimosaic expression in candidate individuals should be confirmed under a two-photon or confocal microscope.
    ***Note:*** Animals injected with lissamine-tagged morpholino can be screened in the same fashion (Figures 9G–9I).

## Expected outcomes

**Figure 10:**
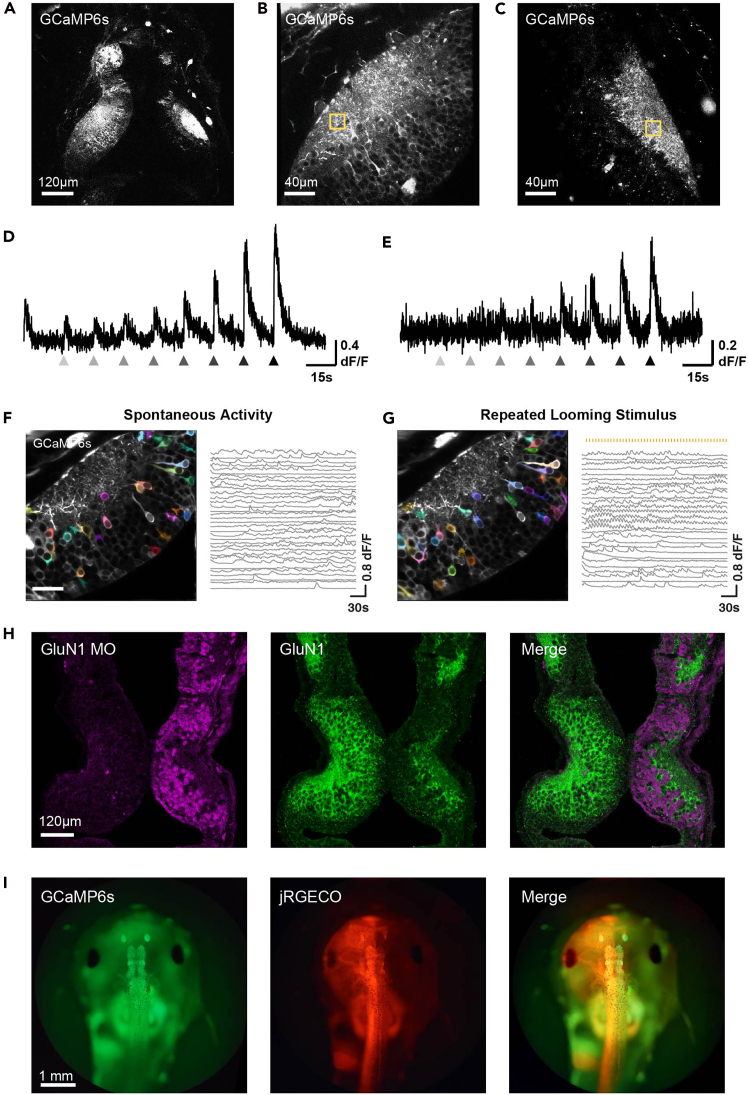
Example results (A) Two-photon optical section from a bilateral hemimosaic GCaMP6s animal. GCaMP florescence can be seen restricted to the left tectum and the neuropil region of the right tectum. (B) Two-photon optical section from the left tectal hemisphere of a bilateral hemimosaic GCaMP6s animal with GCaMP florescence in postsynaptic tectal neurons on the left side. Yellow box marks a region of interest (ROI) in the neuropil area. (C) Two-photon optical section from the right tectal hemisphere of the same animal as shown in (B), displaying GCaMP fluorescence in RGC axon terminals distributed in the neuropil area. Yellow box marks an ROI in the neuropil area. (D) Example visual stimulus-evoked tectal cell calcium response trace, averaged over the ROI marked in (B). Grey triangles under the trace mark timepoints when a stimulus was shown to the right eye (a luminance step from white to various shades of grey, displayed on a small LED screen positioned next to the eye. The colour of the triangles represents the intensity of the luminance step – darker colours represent larger luminance steps). (E) Example visual stimulus-evoked RGC axonal calcium response trace averaged over the ROI marked in (C). The visual stimulus is the same as in (D) but presented to the left eye. (F) Cell body spontaneous calcium activity recorded from a bilateral hemimosaic GCaMP6s tadpole (different animal from B-E). (Left) Two-photon optical section from the left tectal hemisphere. Color overlays: ROIs of spontaneously active tectal neurons, identified by Suite2P software.^23^ Each ROI contains the cell body of a single tectal neuron that showed spontaneous activity. (Right) Spontaneous calcium activity traces extracted by Suite2P, each trace corresponding to a different ROI in the left panel. (G) (Left) Same two-photon optical section as shown in (F), overlaid with ROIs generated from Suite2P based on responses to a repeated looming stimulus (dark circle on a bright background expanding from small to large, displayed on a small LED screen positioned next to the right eye). (Right) Stimulus-evoked response traces extracted by Suite2P, each trace corresponding to a different ROI in the left panel. Top orange ticks denote the onset times of stimulus presentations (once every 6 seconds). (H) Confocal immunofluorescent section of a bilateral hemimosaic GluN1-MO animal, adapted from Kesner et al.^1^ Left: MO-lissamine fluorescence (magenta); middle: immunofluorescence staining for GluN1 (green); right: merged image. (I) Widefield epifluorescence image of a GCaMP6s transgenic tadpole with hemimosaic expression of jRGECO from mRNA blastomere injection. Left: GCaMP fluorescence (green); middle: jRGECO fluorescence (red); right: merged image.

Hemimosaic animals produced from this protocol will show GCaMP6s fluorescence or GluN1 MO lissamine signal in optic tectal cells in one tectal hemisphere and RGC axon terminals in the opposite tectal hemisphere (Figure 10). These hemimosaic animals effectively allow comparisons between pre- and postsynaptic neurons in the retinotectal circuit of the same animal: for GCaMP6s animals, pre- and postsynaptic responses to visual stimuli can be compared (Figures 10B–10E); for GluN1 MO animals, the effects of pre- or postsynaptic GluN1 knockdown on the electrophysiological properties of tectal neurons can be contrasted.^1^

Blastomere injection can be flexibly paired with other labelling techniques to create more powerful hemimosaic models. For instance, we have previously injected jRGECO1a^25^ mRNA into GCaMP transgenic embryos (eggs from albino frogs fertilized with sperm from ElasGFP:Tubb2-GCaMP6s transgenic frogs) to produce tadpoles with pan-neuronal GCaMP6s expression and jRGECO1a expression restricted to one lateral half (Figure 10I), the expression of two differently colored calcium indicators allowing visualization of pre- and postsynaptic circuit components within the same tectal hemisphere.

## Limitations

One technical limitation to the blastomere injection method is its effects often last for a limited period of time. Since mRNA or MO injected into an embryo through blastomere injection will be distributed throughout the animal as it develops, the relative concentration of mRNA or morpholino will eventually degrade or be diluted beyond the level needed to maintain effective GCaMP fluorescence or MO knockdown. This limits the time window within which hemimosaic animals produced from this protocol can be used for experiments to 2–3 weeks post-fertilization.

Compared to transgenic methods, expressing protein via mRNA blastomere injection circumvents the need to breed multiple generations of transgenic animals, offering the advantages of a simpler workflow and shorter turnaround time at the cost of several limitations: In principle, mRNA blastomere injection can be used to express any kind of protein, but from our experience cytoplasmic proteins often produce better results than cell surface proteins. Also, injecting mRNA at an early stage and ubiquitously is not suitable for targeting protein expression to specific cell types.

## Troubleshooting

### Problem 1: Primed female frogs not laying eggs

Typically, frogs injected with hCG in the afternoon or evening of the previous day will start laying eggs in early morning. Ovulating females can be identified by cloaca turning bright red to purple in color. Priming female frogs with the hormone injections in Steps 1–2 reliably induces ovulation, but occasionally frogs will fail to ovulate even after performing both priming injections.

### Potential solution

• Wait a few hours to check the frogs again for ovulation. Some females may take longer to start ovulating than others.
• If the frogs do not show signs of ovulation by afternoon, inject another 400 μl of 1000 IU/mL hCG, and check the frogs for ovulation the next morning.
• An unprimed frog can sometimes be made to spawn the next day with a single hCG injection.
• Putting the females in slightly warmer water may encourage ovulation.
• Do not perform more than two hCG injections on the same frog. If a frog fails to ovulate following a second hCG injection, return the frog to its home tank and allow it to enter the resting period.
• Consider replacing older breeding stock with younger animals if recurring issues arise.

### Problem 2: Low-quality embryos

Low quality sperm or eggs can result in a lower fertility rate and lower embryo survival rate. A good batch of embryos will have fertilization and survival rates of up to 90%, while worse batches will see less than 30% eggs reaching first division and higher rates of embryo death and developmental defects.

### Potential solution

• Ensure primed females are kept in holding tanks with clean water, and change the water whenever fecal discharge and unfertilized eggs start to accumulate.
• Prime multiple females for egg collection if a large volume of eggs is needed.
• Perform a second round of fertilization. The same frog can be squeezed several times to obtain eggs. In our experience, the first batch of eggs is often of poorer quality than the following ones. Leave a rest period of 1.5 h between squeezes.
• When dealing with a batch of eggs with low fertilization rate, perform extra washes with ddH_2_O and 0.1× MBSH as necessary and remove unfertilized and unhealthy eggs.
• Monitor embryos closely for their first division, and separate dividing embryos from non-dividing ones in a timely fashion. In general, even in a batch of eggs with a low fertilization rate, embryos that successfully reach two-cell stage will have a good chance of survival.
• Ensure that the testes are not too old, and store in fresh buffer after use.

### Problem 3: Embryos dying off/developmental defects

Embryo mortality and developmental defects can be caused by a variety of reasons, including overly harsh handling of the embryos during *in vitro* fertilization, incorrect blastomere injection settings, as well as bacterial growth and low oxygen levels in the embryo rearing medium.

### Potential solution

• Ensure blastomere injection pipettes are calibrated to dispense the appropriate volume, with a calibrated injection pressure of ≥15 psi to ensure the tip opening is not too large.
• During blastomere injection, pause between injecting every ∼50 embryos to check the integrity of the injection pipette. Perform a test injection into the Ficoll solution in the injection dish to make sure that fluid is being dispensed, and that the dispensed volume is not noticeably larger compared to the previous test injection. Also verify that the injection pressure displayed on the microinjector has not changed, and there is sufficient gas left in the gas tank.
• Keep close track of your injections to make sure the same embryo is not accidentally injected twice.
• If an embryo is damaged during the injection process (contents of embryo seen leaking out, or embryo starts floating, indicating air was injected due to the injection pipette running out of solution) (Figure 8B), discard the embryo.
• Keep developing embryos in a sufficient volume of rearing solution and avoid overcrowding.
• Check animals daily, clean out any dead embryos and change the rearing solution when it gets dirty.

### Problem 4: Insufficient visibility of GCaMP fluorescence

The basal fluorescence of GCaMP6s is not particularly high, and the distribution of fluorescent protein in an animal can be difficult to ascertain at lower expression levels. This can be an issue when screening animals, especially when trying to determine whether an animal displays complete hemimosaicism.

### Potential solution

• To aid in screening, GCaMP6s mRNA can be co-injected with mRNA for a brighter fluorophore of a different color, such as mCherry. For co-injection with GCaMP6s, mCherry mRNA should be used at a working concentration of 0.125 μg/μl.
• Perform a titration to find the best mRNA working concentrations: perform blastomere injection on a range of different mRNA concentrations and compare the resulting animals for the best expression.
• Weak expression of GCaMP6s protein may also be related to the quality of the mRNA. If the mRNA is contaminated and degraded or the RNA is degrading during the injection process the expression levels will be lower than expected. Make sure to work RNase-free during the whole process to keep your RNA solution clean.

## Resource availability

### Lead contact

Further information and requests for resources and reagents should be directed to and will be fulfilled by the lead contact, Dr. Edward Ruthazer (edward.ruthazer@mcgill.ca).

### Technical contact

Technical questions on executing this protocol should be directed to and will be answered by the technical contact, Anne Schohl (anne.schohl@mcgill.ca).

### Materials availability

This study did not generate any unique reagents.

### Data and code availability

3D print files for the blastomere injection chamber used in this study are available at Github: https://github.com/RuthazerLab/Hemimosaic-3d-models (https://doi.org/10.5281/zenodo.18318247).

## Acknowledgments

The pCS2+ plasmid was created by David Turner (University of Michigan, Ann Arbor, MI, USA). This work was supported by a Brain Canada (BC) Platform Grant to the Canadian Optogenetics & Vectorology Foundry, grants to E.R. from the Canadian Institutes of Health Research (CIHR: FDN-143238; PJT-180478) and the Natural Sciences and Engineering Research Council (NSERC: RGPIN-2024-05306), and fellowships from NSERC CGS-M (V.J.L.), CIHR Vanier CGS-D (P.M.K.), Healthy Brains for Healthy Lives Canada First Research Excellence Fund (D.F.), CIHR/BC/Heart and Stroke Personnel Award for Indigenous Scholars (D.F.), and NSERC PGS-D (D.F.) and NSERC PGS-D (N.B.).

## Author contributions

V.J.L. and P.M.K. performed the experiments, wrote the manuscript, and produced the figures. D.F., A.S., and N.B. performed the experiments, edited the manuscript, and provided images for figures. E.R. edited the manuscript and figures.

## Declaration of interests

The authors declare no competing interests.

## Supplemental information

Methods video S1. Restraining and squeezing a female frog to collect eggs for in vitro fertilization, related to step 17
